# SimCAL: a flexible tool to compute biochemical reaction similarity

**DOI:** 10.1186/s12859-018-2248-5

**Published:** 2018-07-03

**Authors:** Tadi Venkata Sivakumar, Anirban Bhaduri, Rajasekhara Reddy Duvvuru Muni, Jin Hwan Park, Tae Yong Kim

**Affiliations:** 1 Bioinformatics Lab, Samsung Advanced Institute of Technology, Bangalore, 560037 India; 2 Biomaterials Lab, Materials Center, Samsung Advanced Institute of Technology, Gyeonggi-do, 443803 South Korea

**Keywords:** Reaction similarity, Transformation similarity, Similarity measures, Fingerprint

## Abstract

**Background:**

Computation of reaction similarity is a pre-requisite for several bioinformatics applications including enzyme identification for specific biochemical reactions, enzyme classification and mining for specific inhibitors. Reaction similarity is often assessed at either two levels: (i) comparison across all the constituent substrates and products of a reaction, reaction level similarity, (ii) comparison at the transformation center with various degrees of neighborhood, transformation level similarity. Existing reaction similarity computation tools are designed for specific applications and use different features and similarity measures. A single system integrating these diverse features enables comparison of the impact of different molecular properties on similarity score computation.

**Results:**

To address these requirements, we present SimCAL, an integrated system to calculate reaction similarity with novel features and capability to perform comparative assessment. SimCAL provides reaction similarity computation at both whole reaction level and transformation level. Novel physicochemical features such as stereochemistry, mass, volume and charge are included in computing reaction fingerprint. Users can choose from four different fingerprint types and nine molecular similarity measures. Further, a comparative assessment of these features is also enabled. The performance of SimCAL is assessed on 3,688,122 reaction pairs with Enzyme Commission (EC) number from MetaCyc and achieved an area under the curve (AUC) of > 0.9. In addition, SimCAL results showed strong correlation with state-of-the-art EC-BLAST and molecular signature based reaction similarity methods.

**Conclusions:**

SimCAL is developed in java and is available as a standalone tool, with intuitive, user-friendly graphical interface and also as a console application. With its customizable feature selection and similarity calculations, it is expected to cater a wide audience interested in studying and analyzing biochemical reactions and metabolic networks.

**Electronic supplementary material:**

The online version of this article (10.1186/s12859-018-2248-5) contains supplementary material, which is available to authorized users.

## Background

Knowledge of biochemical reaction similarity is important for a wide range of biotechnological applications, such as, classification of enzymes [1–4], identification of missing enzymes in metabolic pathways [5, 6], identification of promiscuous enzymes in understanding the metabolic network evolution [7] and mine specific reaction substrates and the inhibitors [8–11]. Similarity between chemical reactions, referred to as reaction similarity, can be calculated at multiple levels: Transformation level similarity is computed by considering only the atoms and bonds that are undergoing transformation, at different degrees of neighborhood information [12]. Reaction level similarity considers molecular information of the entire substrates and products constituting a biochemical reaction [13]. Assessing reaction similarity as transformation level enables classification of enzyme function based on reaction mechanism [14–16]. Evaluating similarity at reaction level assists novel pathway engineering by identifying possible native target molecules in organisms and relevant possible enzymes that can catalyze novel steps [17–19].

Depending on the objective, reaction similarity computations rely on different feature representations to achieve required purposes. RxnFinder [20], a reaction search engine tool, uses Reaction Difference Fingerprint (RDF) for finding similar reactions. RDF is the difference between the union of features collected on substrate side and product side of a reaction. Unlike RDF, which is a fingerprint based representation of differences, RDM (reaction center, difference atom and matched atom) pattern [21] is a non-fingerprint based representation of transformation region. An extension of the RDM pattern is used in Metabolite and Reaction Inference based on Enzyme Specificities (MaRIboES) [22] for identifying specificity of an enzyme to catalyze a given metabolite or reaction. SimIndex (SI) and SimZyme [5] use two dimensional chemical fingerprints for computing chemical similarity for identifying new enzymatic connections in the metabolic networks. EC-BLAST [23] performs similarity searches using three different techniques, namely, bond changes (BC), reaction centers (RC) and substructure similarity to search and compare enzymatic reactions. Enzyme promiscuity based on reaction similarity is studied using molecular graph descriptors (molsig) [24] . Numerous additional methods aiming to quantify molecular or reaction similarity are reported in literature [25, 26]. From these perspectives it is evident that, computed reaction similarity results are dependent on factors such as the final objective, nature of data, choice of similarity measure and the fingerprint. Hence, obtaining consensus is challenging [27–30] (S1). Thus, it is imperative to customize the assessment in accordance with the application.

An integrated system enabling a combination of various similarity computation approaches along with a choice of features and comparative assessment of results would be of immense help. This article presents SimCAL, a robust tool that allows users to customize the reaction similarity assessment and evaluation in accordance with the desired application. The tool offers flexibility around the selection of different feature types and approaches to compute, compare reaction similarity.

## Implementation

**Fig. 1:**
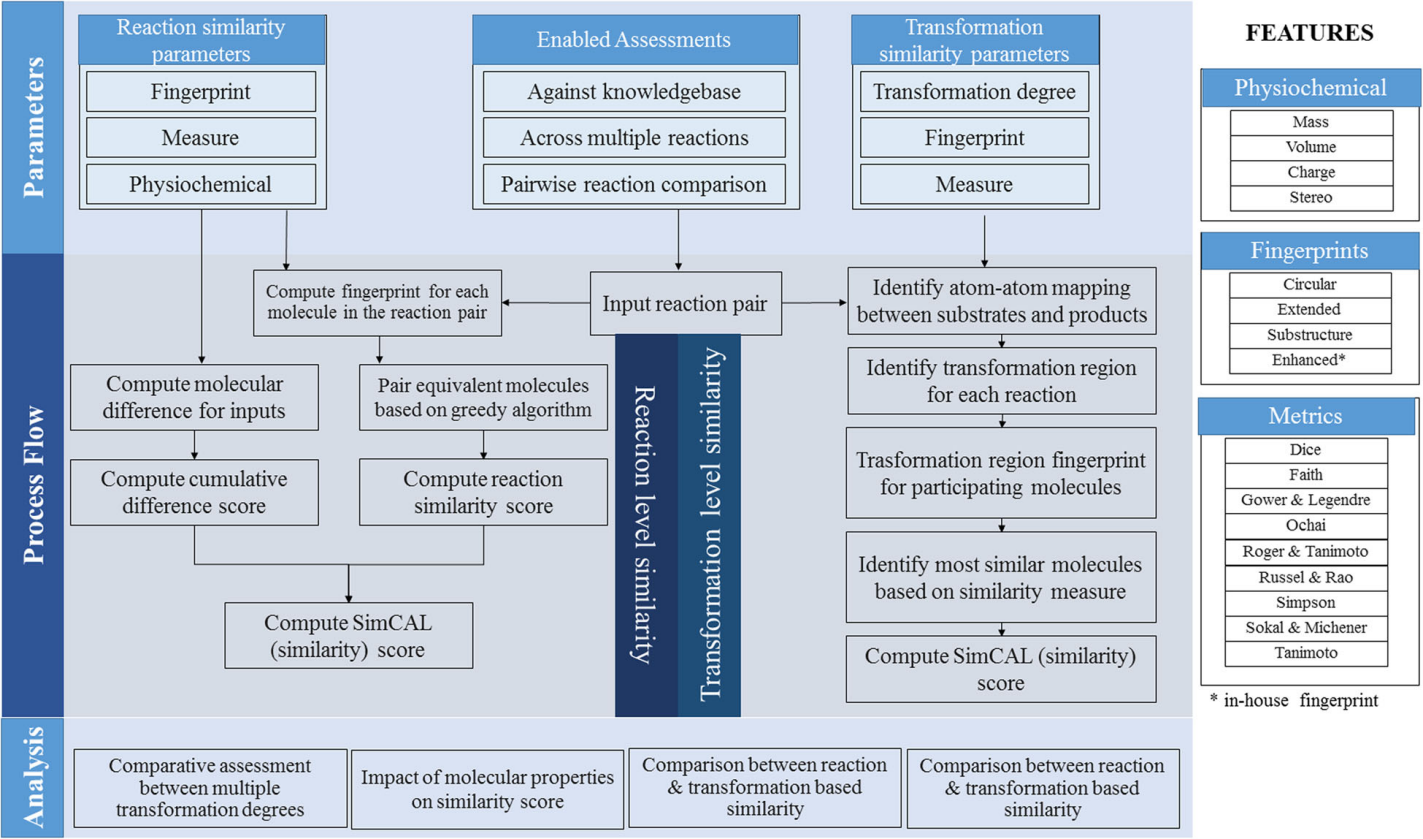
Overview of SimCAL system and list of available features

SimCAL is available both as a user-friendly graphical interface tool and a console application. It is developed in Java (ver 1.7) and uses chemoinformatics routines of Chemical Development Kit, CDK [31] for processing. The key modules of SimCAL are (i) parameter selection, (ii) process flow and (iii) analysis. These are described in Fig. 1. Parameter selection component enables the user to select different features along with the similarity type to be computed using the reaction data provided by the user. Process flow component, provides details of the steps involved in finding reaction similarity at the reaction and transformation levels. Analysis component provides user with several options to perform comparative assessments.

### Parameter selection

Parameter selection module allows selection of different features and their representation that will be used to compute reaction similarity. User begins the analysis by selecting either or both of the reaction similarity types, namely, reaction level and transformation level. This is followed by the selection of any or all of the four fingerprints available in SimCAL, namely, (i) Circular, (ii) Extended, (iii) Substructure and (iv) Enhanced fingerprint. Details of these fingerprints are provided in Table 1. Enhanced fingerprint is an in-house developed improvisation of the extended fingerprint. In enhanced fingerprint, in addition to the molecular descriptors defined through specific binary bits, distinct signatures for charge and stereochemistry are encoded. Further, user can select any or all of the nine similarity measures for computing reaction similarity. The details of similarity measures, are provided in Table 2. The similarity measure calculations are computed using four variables: ***a***, ***b***, ***c*** and ***d***. These variables capture the presence and absence of specific descriptors across two fingerprint vectors A and B related to the two molecules under consideration. ***a*** is the count of set bits in both fingerprint of molecule A and B. ***b*** is the count of set bits in fingerprint of molecule A and not in B. ***c*** is the count of set bits in fingerprint of molecule B and not in A. ***d*** is the count of unset bits in both the fingerprints of the molecules A and B. The size of a fingerprint is given by ***n*** = (***a*** + ***b*** + ***c*** + ***d***). The default selection measure used in SimCAL is Tanimoto. Reaction similarity calculations are further adjusted by considering variance of specific molecular properties such as mass, volume [32] and pH based charge calculations. Impact of the parameters (reaction similarity type, fingerprint, molecular properties, and measure) is highlighted using a simple dataset as discussed in Additional file 1: (S2, S3).

**Table 1:** List of four fingerprints available in SimCAL

S. No.	Name	Description
1.	Circular Fingerprint	Circular fingerprint is based on CDK’s [31] circular fingerprinter and is functionally equivalent to ECFP-2 [43]
2.	Extended Fingerprint	Functionally equivalent to ExtendedFingerprinter of CDK [31]. This fingerprint is unique from the standard form since it accounts for ring systems. Default length size is 1024 bits.
3.	Substructure Fingerprint	This is a structural key type fingerprint which considers assessment of 307 different substructures and is based on KlekotaRothFingerprinter [44] in CDK.
4.	Enhanced Fingerprint	An in-house developed improvised extended fingerprint which accounts for stereochemistryand charges on molecules.

**Table 2:** List of binary similarity measures included in SimCAL

S. No.	Measure	Definition	Range
1.	Tanimoto	$\frac{a}{\left(a+b\right)+\left(a+c\right)-c}$	[0-1]
2.	Dice	$\frac{2a}{2a+b+c}$	[0-1]
3.	Ochiai	$\frac{a}{\sqrt{\left(a+b\right)}\left(a+c\right)}$	[0-1]
4.	Simpson	$\frac{a}{\min \kern0.5em \left(a+b,a+c\right)}$	[0-1]
5.	Russell and Rao	$\frac{a}{a+b+c+d}$	[0-1]
6.	Sokal and Michener	$\frac{a+d}{a+b+c+d}$	[0-1]
7.	Faith	$\frac{a+0.5d}{a+b+c+d}$	[0-1]
8.	Gower and Legendre	$\frac{a+d}{a+0.5\left(b+c\right)+d}$	[0-1]
9.	Roger and Tanimoto	$\frac{a+d}{a+2\left(b+c\right)+d}$	[0-1]

The measures are in correspondence to [45]. *a* is count of set bits in both fingerprint of both the molecules. *b* is count of set bits in fingerprint of first molecule and not in second molecule. *c* is count of set bits in fingerprint of second molecule and not in first molecule. *d* is count of unset bits in both fingerprint of both the molecules. The size of the fingerprint is given by *n* = (*a* + *b* + *c* + *d*)

### Process flow

SimCAL facilitates the computation of reaction similarity score based on transformation regions [25] and whole reaction level [7, 23, 33].

#### Similarity score computation: Transformation region based

**Fig. 2:**
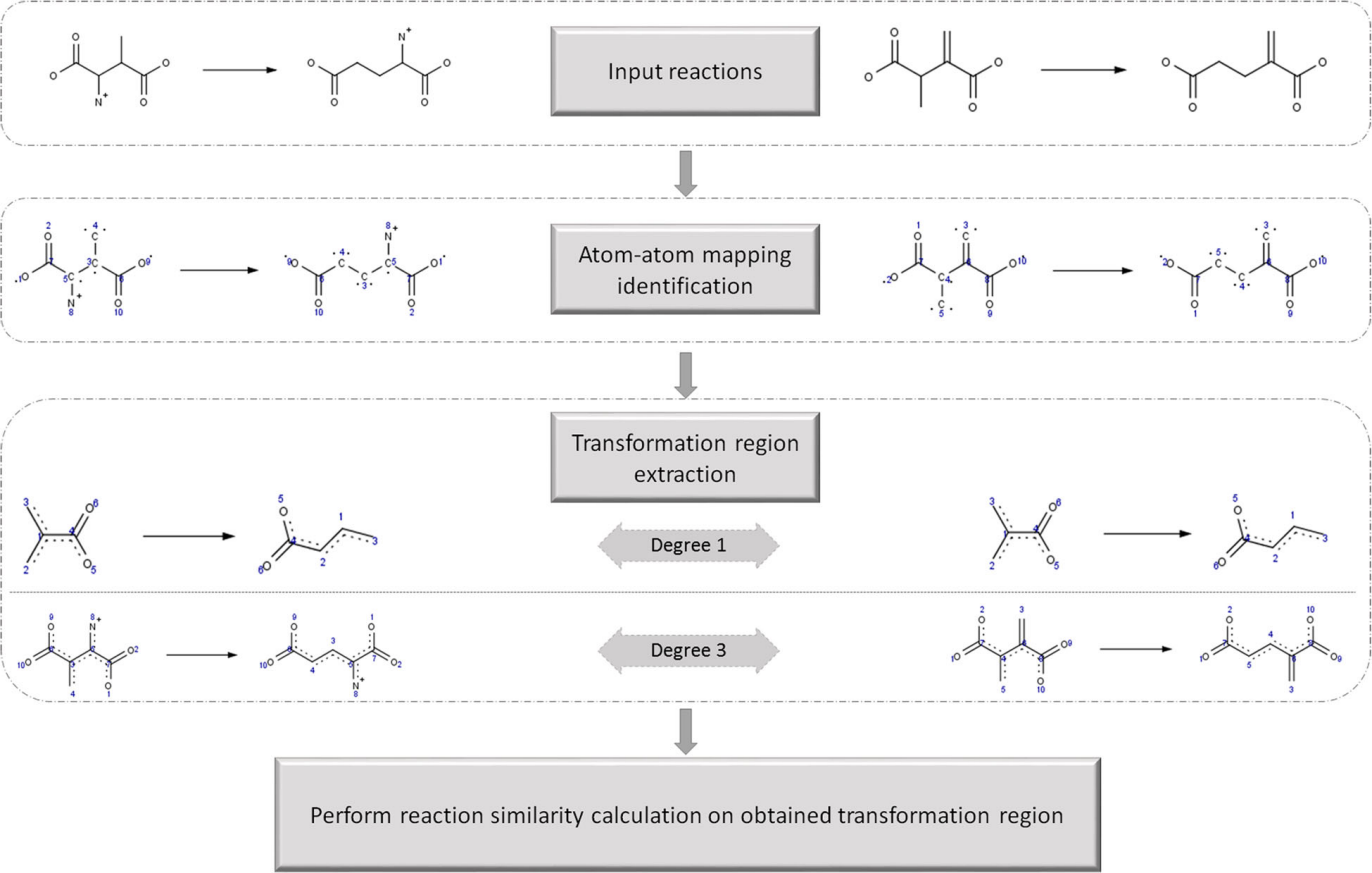
Exemplary computation of transformation based similarity

Transformation region in a chemical reaction comprises of the reaction center (sets of atoms across the molecules undergoing bond rearrangement) and its neighborhood. The extent of the neighborhood defining the transformation region is captured through the transformation degree [34]. For example a transformation degree of one (which is default and can be defined by user) would comprise the reaction center and all atoms associated with the reaction center at one bond distance. The transformation region from a reaction is extracted based on the atom-atom mapping. The atom-atom mapping can either be provided by the user or calculated using reaction decoder tool (RDT) [35]. The extracted transformation region is further processed using the user selected fingerprint and measure to compute the reaction similarity using the reaction level similarity calculation procedure. Process outline for the computation of transformation similarity is shown in Fig. 2.

#### Similarity score computation: Whole reaction level

**Fig. 3:**
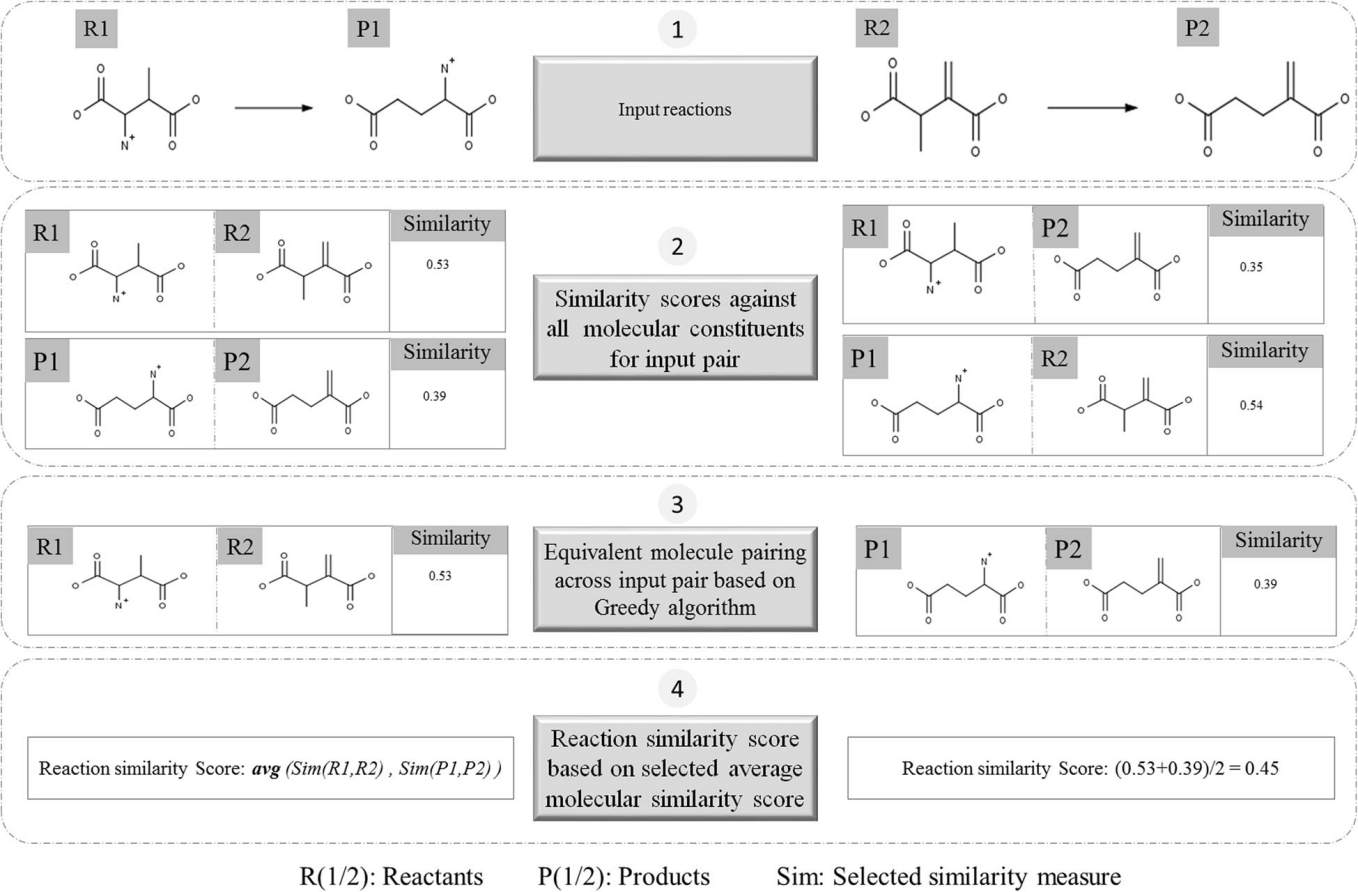
Schematic processing of the fingerprint based similarity computation

The computation of whole reaction level similarity considers all substrates and products in a reaction to the entirety. All constituent molecules in the reaction are represented in a reaction fingerprint vector. The fingerprints or molecule descriptors vary for different fingerprint methods. This conversion is performed for each input reaction. A greedy algorithm is used to pair molecules across the reactions [13]. The objective of the pairing is to maximize user selected similarity measure. The reaction similarity score is the average of the molecular similarity [13] computed for all equivalent pairs of molecules. Any unpaired molecules are dropped from computations. A schematic of the processing is depicted in Fig. 3.

#### Similarity computation: Molecular property correction

A general constraint of reaction similarity calculation methods is that they do not consider deviation of physicochemical attributes of the constituent molecules in the reaction pair. This can result in erroneous computation of similarity scores. Changes in pH influences the charge of constituent molecules in a reaction, affecting its transformation feasibility. SimCAL provides flexible options for considering four molecular properties viz., stereochemistry, charge, mass and volume in the computation of similarity between reactions. Stereochemistry and molecular charge of constituent molecules of a reaction are assessed using the circular fingerprinter [31] and an in-house developed enhanced fingerprint. Since they are represented as bits within the fingerprint vector, their impact is accounted for while computing the reaction similarity score using a selected measure (Fig. 3). The impact of environment such as pH on a chemical transformation is well documented [36]. SimCAL accepts a user defined pH value (default 7) to compute theoretical pKa of input molecules [37] and report the charge on the constituent molecules. Based on the reported charge distribution on constituent molecules, the in-house developed enhanced fingerprint is then used to compute reaction similarity.

SimCAL computes the molecular mass and the molecular volume of the constituents of the reaction as implemented in CDK. The variability associated with mass and volume between the paired molecular entities are computed using a generalized Jaccard distance [38]. The computed average Jaccard distance along with the reaction fingerprint based similarity score is used to compute the final reactions similarity (Eq. 1).

1 $$ {R}_s\kern0.5em =\kern0.5em \raisebox{1ex}{${R}_f$}\!\left/ \!\raisebox{-1ex}{$1+{J}_{dist}$}\right. $$

where *R*_*s*_ = Reaction similarity score, *R*_*f*_= Reaction similarity score based on fingerprint and *J*_*dist*_= Variation of molecular property obtained through Jaccard distance *J*_*dist*_ is the average Jaccard distance, Eq. (2). This is obtained from the generalized Jaccard score (***J***_***s***_), Eq. (3) for *N* paired molecules in the under study reaction pairs. Each equivalent pair of molecules is represented by *a*, and *b*. The Jaccard distance *J*_*dist*_ may be computed for the selected properties based on the selection of a user (mass, volume or both).

2 $$ {J}_{dist}=\raisebox{1ex}{$1-\sum \left({J}_s\right)$}\!\left/ \!\raisebox{-1ex}{$N$}\right. $$

3 $$ {J}_s=\raisebox{1ex}{$\min \left(a,b\right)$}\!\left/ \!\raisebox{-1ex}{$\max \left(a,b\right)$}\right. $$

### Analysis

The analysis enables user to further customize and assess similarity calculations through comparative assessment. SimCAL provides three types of comparative assessment techniques: (i) transformation degree comparative assessment, (ii) fingerprint comparative assessment and (iii) similarity measure comparative assessment. Transformation degree based assessment provides transformation level based similarity by considering different degrees of user selected neighborhood length. Fingerprint based comparative assessment can be used to compare the results obtained from different fingerprints the user has selected. To compare reaction similarity results of chosen molecular similarity measures, similarity measure comparative assessment can be used. All these comparative assessments can be performed at both reaction level as well as transformation level. Once a simulation is completed on user provided data, SimCAL provides a unique feature to either select entire set of reactions or a subset of results to re-evaluate them using other parameter selection.

## Results & discussion

### SimCAL feature evaluation

As per the four digit Enzyme Commission (EC) nomenclature, two reactions are said to be similar if the enzymes catalyzing those reactions are identical up to the 3rd level (sub-subclass) [39].Reaction pairs catalyzed by enzymes having EC number until the first 3 digits were classified similar (true positive), while others where annotated as not similar (true negative). Using this hypothesis, we evaluated the performance of SimCAL to compute reaction similarity with the following parameters:

- Transformation similarity (degree 1)
- Reaction similarity based on extended fingerprint
- Reaction similarity based on enhanced fingerprint (considers charge and stereo-centers)
- Reaction similarity based on enhanced fingerprint and molecular properties (mass and volume)

**Fig. 4:**
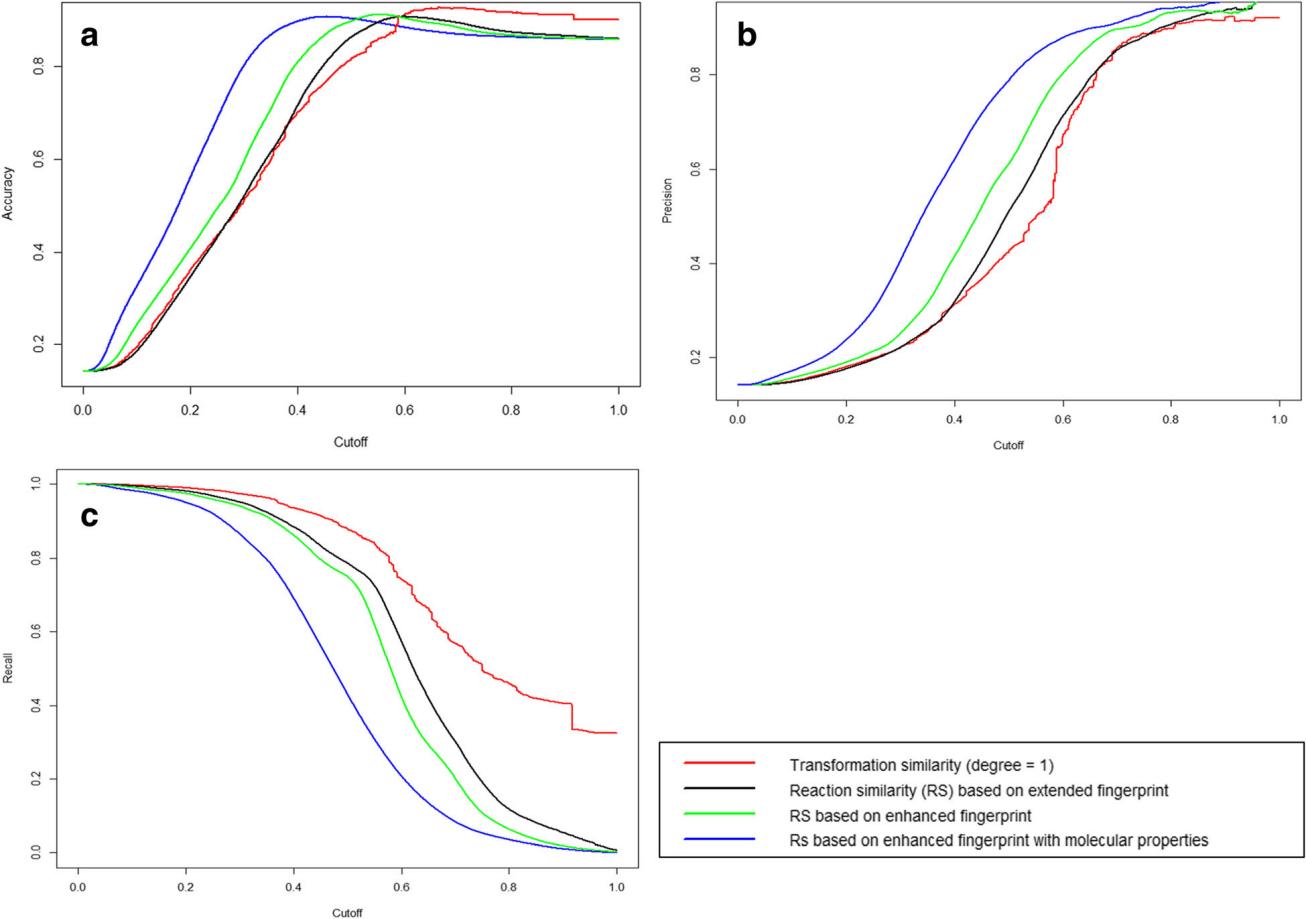
Receiver operating curves (ROC) for various approaches. **a** Reports the dependency of accuracy of predicting similar and non-similar reactions with cutoff (threshold) using the various approaches. **b** Reports the dependency of precision of predicting similar and non-similar reactions with cutoff (threshold) using the various approaches. **c** Reports the dependency of recall (true predictive rate) of predicting similar and non-similar reactions with cutoff (threshold) using the various approaches

The dataset comprised of 3,688,122 reaction pairs obtained by pairing (all-against-all) reactions from MetaCyc [40] within each EC class. The prediction performance was accessed using receiver operator characteristic (ROC) as implemented in the ROCR package [41]. The Area under the curve (AUC), that estimates the robustness of the method, calculated for the above four parameters are as follows: 0.92, 0.89, 0.90 and 0.90. The performance of different ROC properties trends against a threshold score (cutoff) is plotted in Fig. 4b. The prediction of the accuracy of the methods are provided in Fig. 4a. The accuracy of reaction similarity based on enhanced fingerprint and molecular properties has the best accuracy, which also has higher precision value as shown in Fig. 4b. The recall plot on the other hand suggests that the transformation similarity based approach performs better. The ROC experiments suggest that the reaction similarity obtained by using enhanced fingerprints and molecular properties outperforms other approaches.

### Benchmarking over existing methods

Further SimCAL’s performance is benchmarked against two existing methods EC-BLAST [23] and the molecular signature based reaction similarity method [24]. For benchmarking study we consider the molecular signature based reaction chemical similarity method [24] (with h set to 4) and all the three approaches provided in EC-BLAST [23]. Along with these, three features considered from SimCAL are transformation level similarity with degree 1, reaction level similarity using extended fingerprint and enhanced fingerprint along with molecular property variance. It should be noted that SimCAL uses bit based fingerprints whereas the two tools against which it is compared consider count based fingerprint for their assessment.

**Fig. 5:**
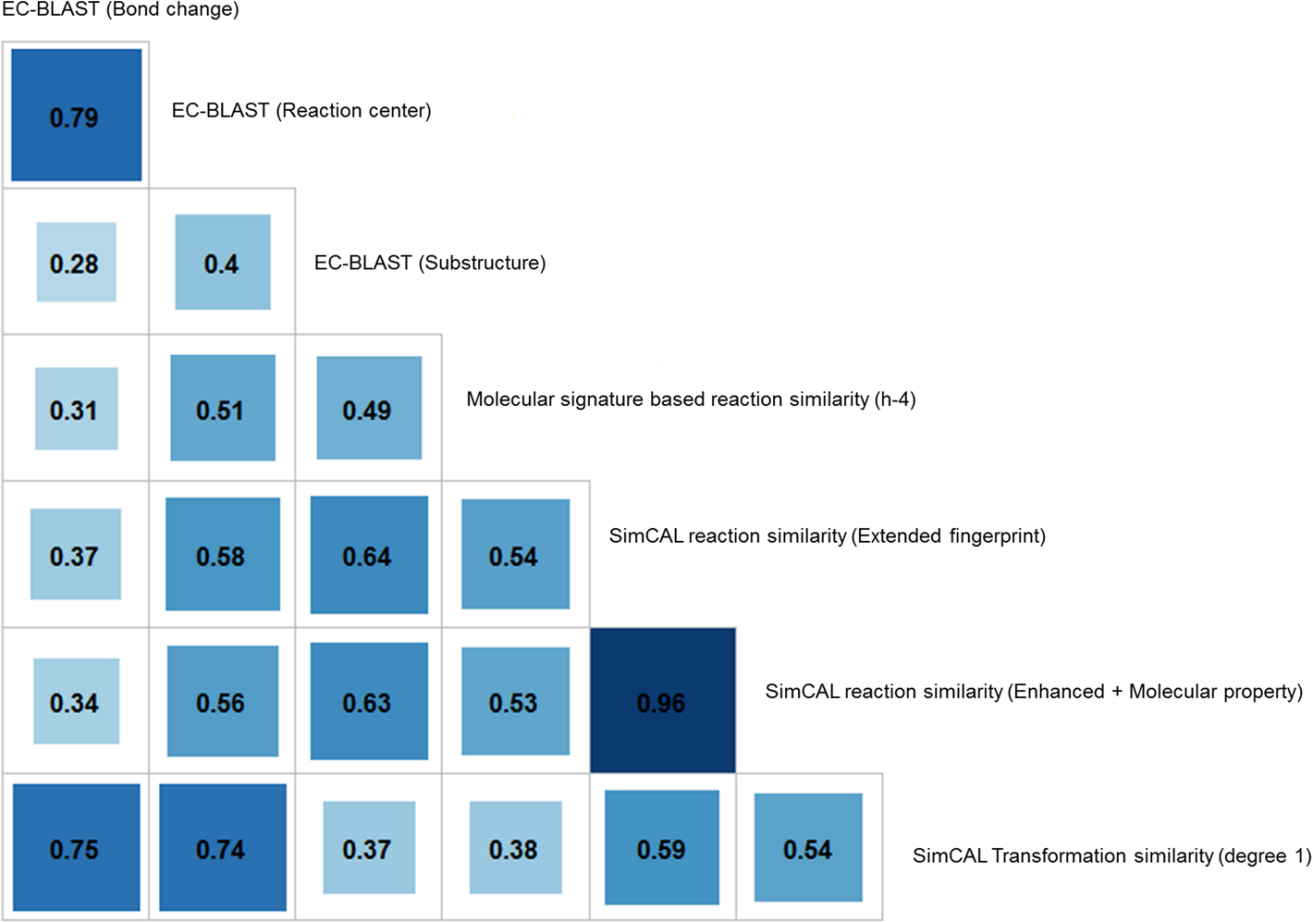
Correlation matrix across the various approaches within SimCAL and 3 approaches of EC-BLAST and the molecular signature based chemical similarity method

The same dataset used for SimCAL feature evaluation is used for benchmarking as well. Pearson correlations of the results between approaches are summarized in Fig. 5. The intensity of color in the box is directly proportional to the correlation between any two methods under consideration. The correlation analysis shows that results obtained by EC-BLAST (bond change), EC-BLAST (reaction center) and SimCAL transformation similarity are well correlated among each other with minimum value of 0.74 and maximum of 0.79. SimCAL (extended fingerprint) (0.54) is correlated slightly higher to the molecular signature based reaction similarity than EC-BLAST (reaction center) (0.51). Both SimCAL (extended fingerprint) and SimCAL (enhanced fingerprint + molecular property) show a very high correlation of 0.96. This is due to the fact that the dataset contains very few reactions, catalyzed by the same enzyme class up to 3rd digit have differences in stereo or charge or molecular property variance. It was observed that the approaches at a large scale shares moderate to strong correlation [42].

## Conclusion

The identification of reaction similarity has a growing range of applications in biochemistry. SimCAL, the integrated tool presented here, enables reaction similarity computation at different levels with a wide choice of feature selection and comparative assessment of final results. The reaction similarity computation is further enhanced by using additional molecular properties, stereo and charge specific information. It is believed that the tool will cater to a wide audience in the field of biochemistry and metabolic engineering.

## Availability and requirements

**Project Name**: SimCal.

**Project home page**: https://sourceforge.net/projects/simcal/

**Operating systems**: Windows, Linux and Mac.

**Programming language**: Java.

**Other requirements**: Java 1.7 or higher.

**License**: LGPL.

Data generated and analyzed during the current research is available in the supplementary data files, along with the R scripts.

## Additional file

Additional file 1: Supplementary material. (DOCX 1098 kb)

## Abbreviations

AUC: Area under the curve
CDK: Chemistry development kit
RDT: Reaction decoder tool
ROC: Receiver operator characteristics
