# Supplementary material for: SimCAL: a flexible tool to compute biochemical reaction similarity
Source: BMC Bioinformatics. 2018 Jul 3;19:254. doi: 10.1186/s12859-018-2248-5 (PMC6029250; doi:10.1186/s12859-018-2248-5)
Supplement: Supplementary file 1 — Supplementary material. (DOCX 1098 kb) [file 12859_2018_2248_MOESM1_ESM.docx]

Supplementary Material

Contents

[S1 Reaction similarity and dependency 2](#_Toc488406296)

[S2 SimCAL: Flexibility and Processing 6](#_Toc488406297)

[S3 SimCAL: Impact of features 8](#_Toc488406298)

[S4 References 10](#_Toc488406299)

**List of Figures**

[Figure S1: Pearson correlation of computed reaction similarity scores using measures, Tanimoto, Simpson and Gower and Legendre coefficient. The correlation was computed for 6 sets of 40 reactions selected. Each dataset were from separate EC classes. Extended fingerprint [10] is used to represent the constituents molecules of the reactions. 3](#_Toc470703127)

[Figure S2 Pearson correlation of computed reaction similarity scores using measures, Tanimoto, Simpson and Gower and Legendre coefficient. The correlation was computed for 6 sets of 40 reactions selected. Each dataset were from separate EC classes. Circular fingerprint [11] is used to represent the constituents molecules of the reactions. 4](#_Toc470703128)

[Figure S3 Pearson correlation of computed reaction similarity scores using measures, Tanimoto, Simpson and Gower and Legendre coefficient. The correlation was computed for 6 sets of 40 reactions selected. Each dataset were from separate EC classes. Substructure fingerprint [10] is used to represent the constituents molecules of the reactions. 5](#_Toc470703129)

[Figure S4: Schematic flow of similarity computation in SimCAL system 8](#_Toc470703130)

**List of Tables**

[Table S1: List of 4 fingerprints available in SimCAL (user interface) 6](#_Toc470703131)

[Table S2 List of binary similarity measures included in SimCAL (user interface). The measures are in correspondence to [13]. Tanimoto coefficient measure is set of the default measure. 6](#_Toc470703132)

[Table S3: Similarity scores on based on various features embedded in SimCAL. Based on the application the user may choose a set of features that may be critical for accurate assessment of similarity. Ability of an enzyme to catalyze a pairs of reaction is considered as an application is the current assessment. 9](#_Toc470703133)

# Reaction similarity and dependency

It is observed that often deriving consensus across methods is challenging [1]. This may be due to multiple factors. Some factors are owing to the molecular descriptors (fingerprints) used to represent constituent molecules and the quantifying similarity measures [2–7]. Moreover nature of the data being analyzed could also play a role [8]. Similar data dependency is expected within the biochemical space.

Influence of data nature on biochemical reactions similarity computation was explored. The experiment was performed on 6 reaction datasets. Each of the data set comprised of 40 randomly selected reactions corresponding to a single EC class. Reaction fingerprints were constructed based on the molecular descriptors (fingerprints) of the constituent molecules to represent the reactions. An all-against all pairwise reaction similarity scores for each set were computed [9]. Tanimoto, Simpson and Gower and Legendre coefficients were used as measures to compute the similarity score. Influence of skewed representation of molecules was neutralized by using 3 different molecular descriptors that represents molecules using different features (Extended fingerprint, circular fingerprint and substructure fingerprint).

Consistency in the Pearson correlation across the measures (Tanimoto, Simpson and Gower and Legendre coefficients) was not observed across the enzyme commission classes (Figure S1, Figure S2 and Figure S3). Similar inconsistency was also observed when different molecular descriptors (Extended fingerprint, circular fingerprint and substructure fingerprint) representing the constituent molecules were used. Thus it is being concluded that the inconsistency reported in the molecular similarity and chemical reaction similarity is also existent within the biochemical space.


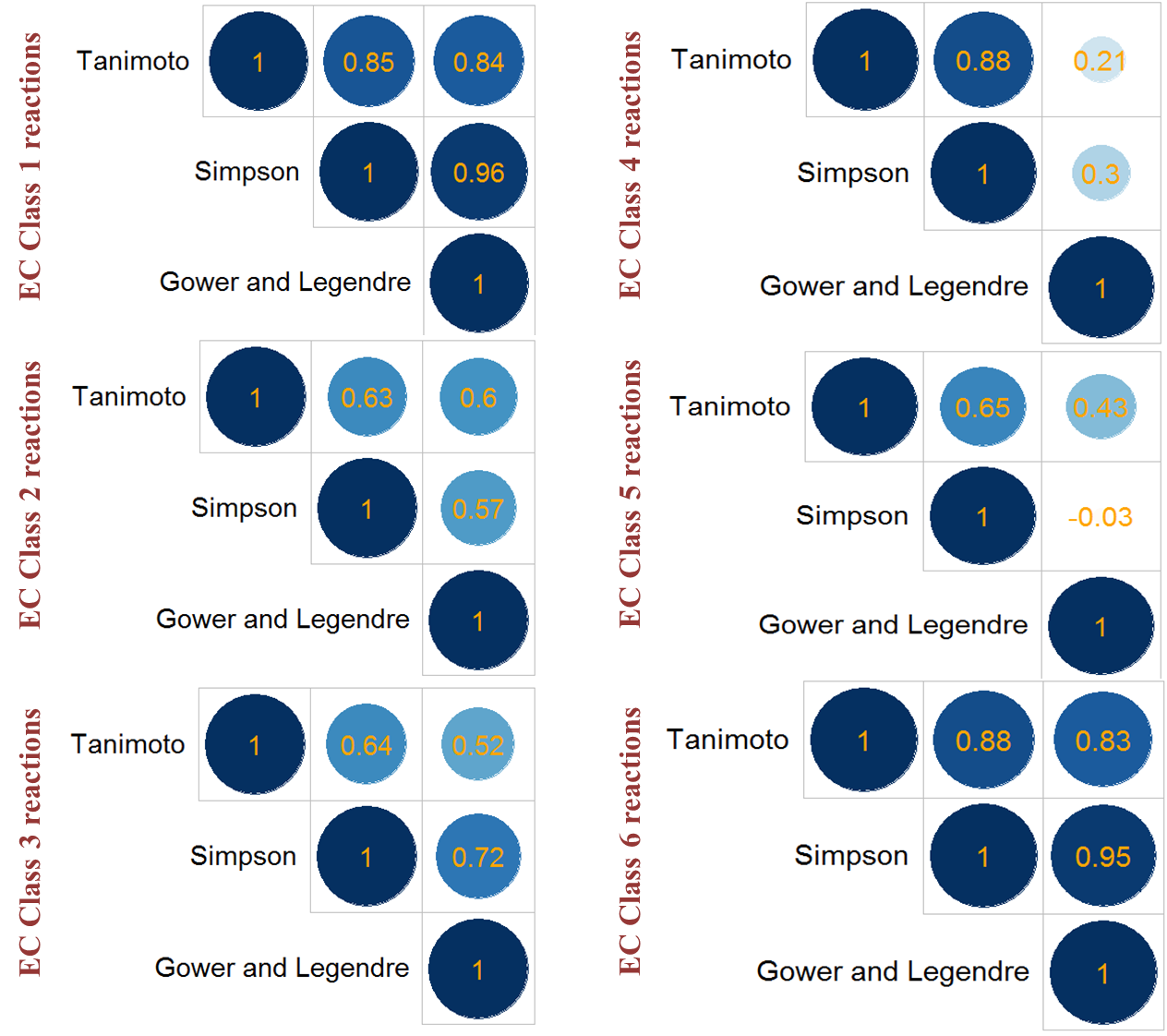


Figure S1: Pearson correlation of computed reaction similarity scores using measures, Tanimoto, Simpson and Gower and Legendre coefficient. The correlation was computed for 6 sets of 40 reactions selected. Each dataset were from separate EC classes. Extended fingerprint [10] is used to represent the constituents molecules of the reactions.


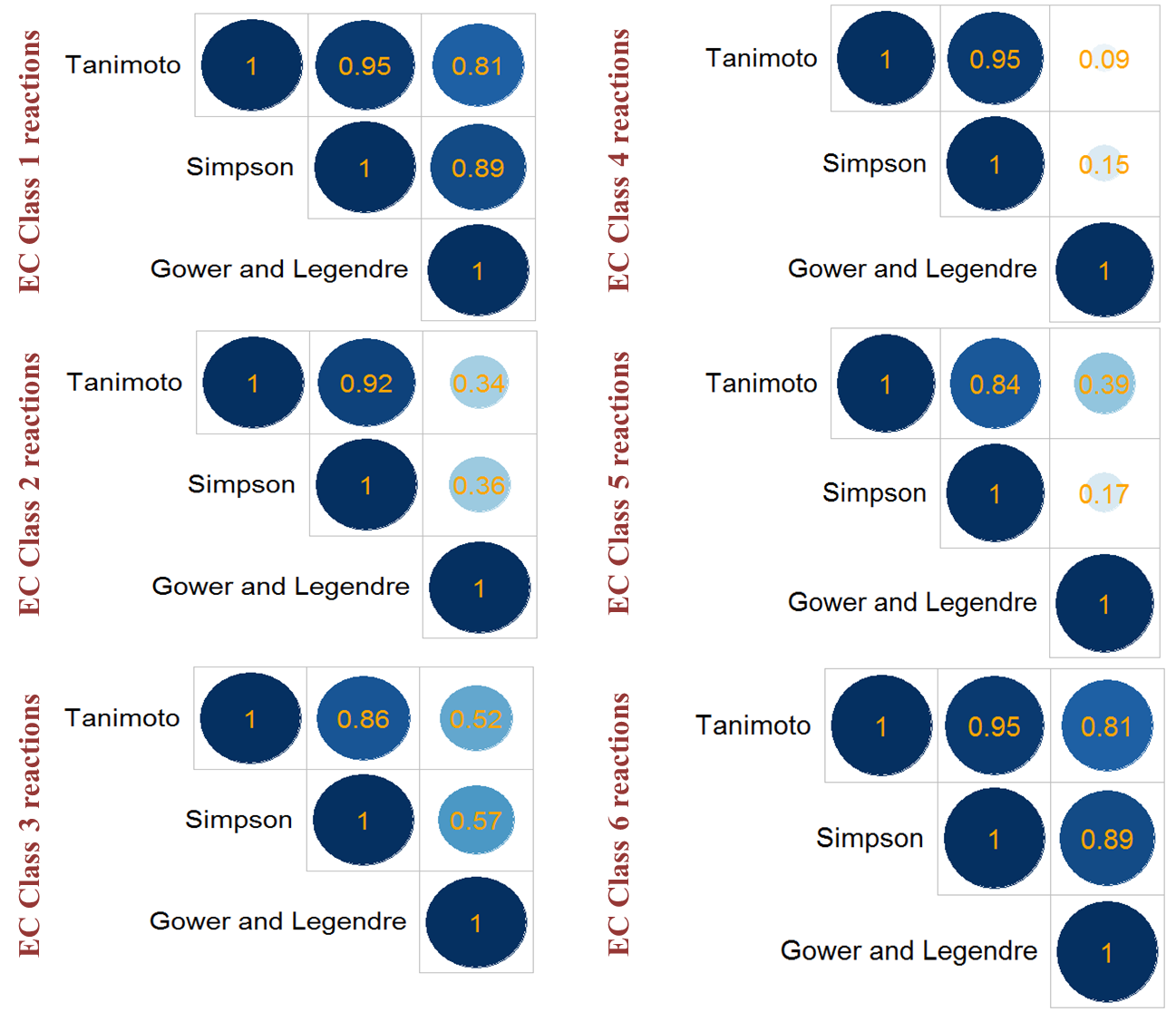


Figure S2 Pearson correlation of computed reaction similarity scores using measures, Tanimoto, Simpson and Gower and Legendre coefficient. The correlation was computed for 6 sets of 40 reactions selected. Each dataset were from separate EC classes. Circular fingerprint [11] is used to represent the constituents molecules of the reactions.


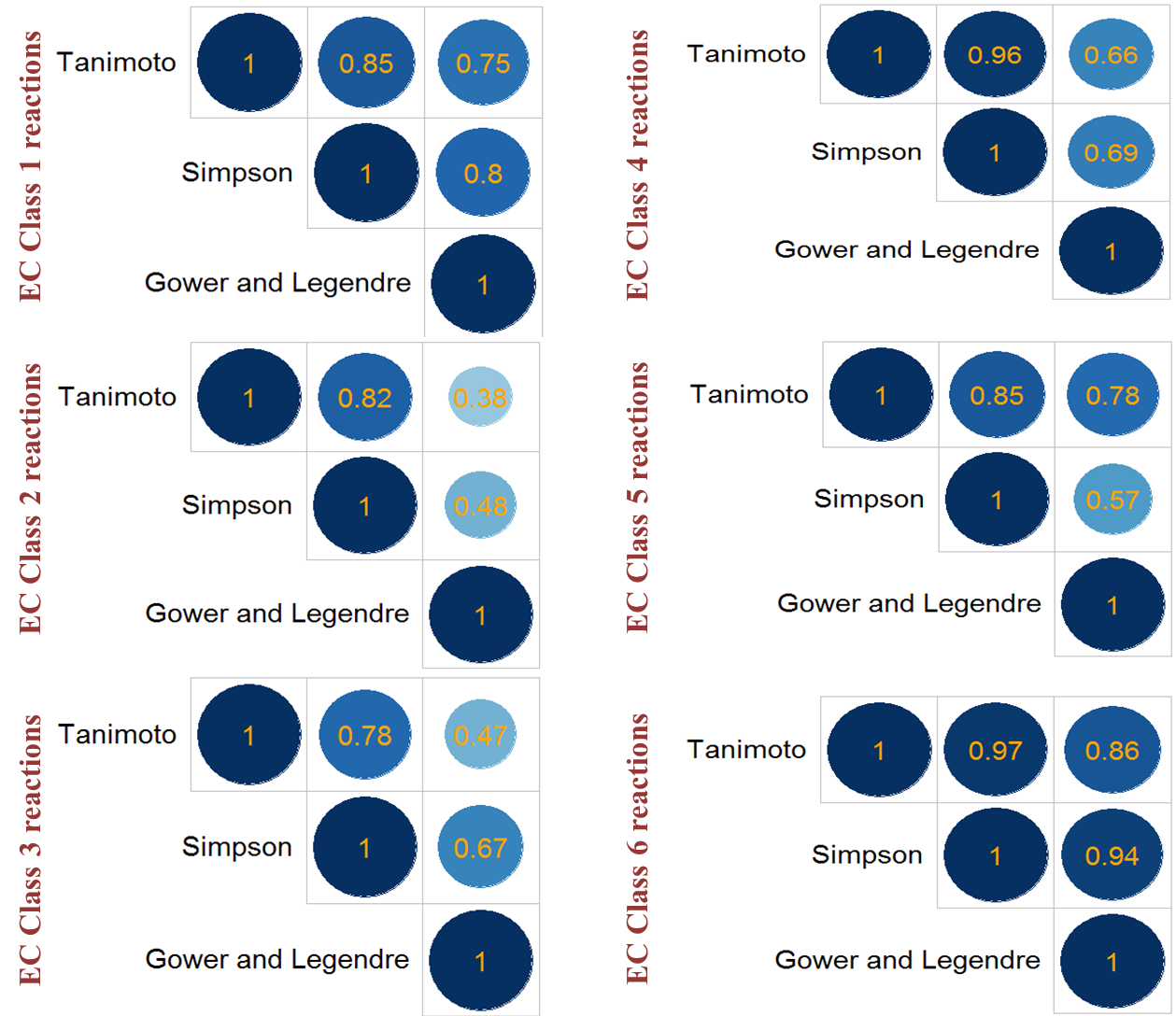


Figure S3 Pearson correlation of computed reaction similarity scores using measures, Tanimoto, Simpson and Gower and Legendre coefficient. The correlation was computed for 6 sets of 40 reactions selected. Each dataset were from separate EC classes. Substructure fingerprint [10] is used to represent the constituents molecules of the reactions.

# SimCAL: Flexibility and Processing

SimCAL is a flexible system where features and parameters may be set based on a specific application. A schematic workflow capturing customizable parameters and features is being reported below (Figure S4).


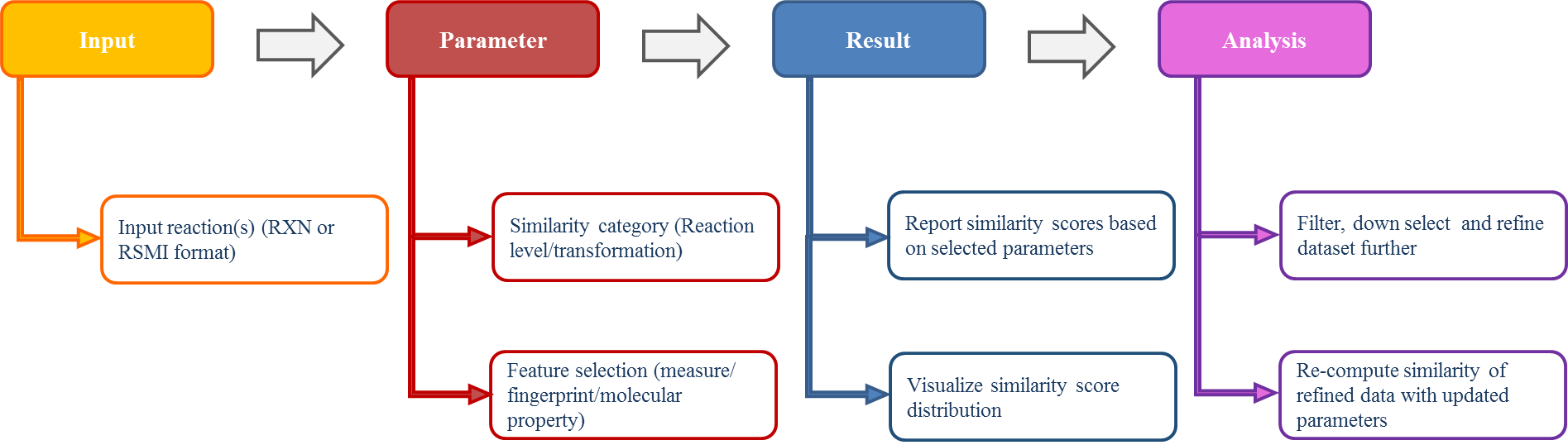


Figure S4: Schematic flow of similarity computation in SimCAL system

# SimCAL: Impact of features

Given an application (enzyme screening, reaction classification or database mining) specific features is better equipped to assess similarity. These features are configurable within SimCAL. Impact of the parameters is highlighted through a sample dataset below (Table S3).

Table S3: Similarity scores on based on various features embedded in SimCAL. Based on the application the user may choose a set of features that may be critical for accurate assessment of similarity. Ability of an enzyme to catalyze a pairs of reaction is considered as an application is the current assessment.

| **Case Study** | **Reaction Pairs** | **Similarity Scores** | **Remarks** |
| --- | --- | --- | --- |
| 1 | 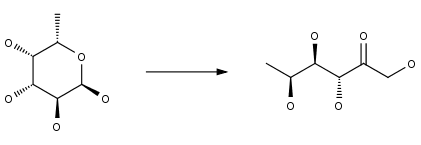 | *Reaction similarity:*  Extended Fingerprint (only): 1  Enhanced Fingerprint (mass): 0.92  Enhanced Fingerprint (Volume): 0.89  Enhanced Fingerprint (Stereo):0.63  *Transformation similarity score:*  Degree 1 :1 | The pair of reactions report high similarity based on most of the features, except when stereo center is considered as a feature. These reaction exhibit differential enzymes catalysis (EC numbers 5.3.1.15 and 5.3.1.25 respectively) |
|  | 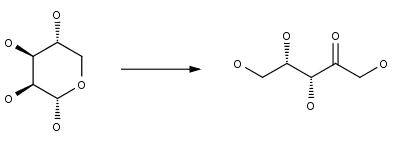 |  |  |
| 2 | 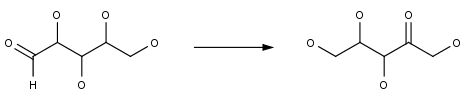 | *Reaction similarity:*  Extended Fingerprint (only): 0.94  Enhanced Fingerprint (mass) : 0.77  Enhanced Fingerprint (Volume): 0.84  Enhanced Fingerprint (Charge): 0.49  *Transformation similarity score:*  Degree 1 :1 | The pair of reactions report same transformation with a salt in one case and the neutral carboxylic acid in the other. The reactions exhibit differential enzymes catalysis (EC numbers 5.3.1.12 and 5.3.1.20 respectively) |
|  | 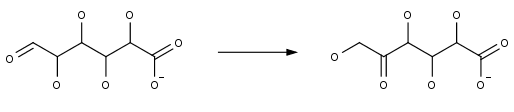 |  |  |
| 3 | 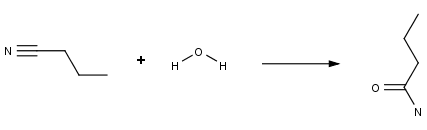 | *Reaction similarity:*  Extended Fingerprint (only): 0.46  Enhanced Fingerprint (mass) : 0.38  Enhanced Fingerprint (Volume): 0.42  Enhanced Fingerprint (Charge): 0.46  *Transformation similarity score:*  Degree 1 :1 | The pair of reactions report same transformation catalyzed by same enzyme 4.2.1.84. The reaction similarity varies between both the reactions along with mass and also volume variability. |
|  | 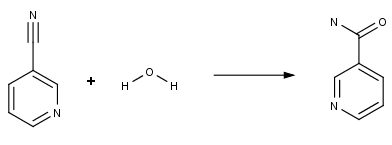 |  |  |

# References

1. Bender A. How similar are those molecules after all? Use two descriptors and you will have three different answers. Expert Opin. Drug Discov. England; 2010;5:1141–51.

2. O’Boyle NM, Sayle R. Comparing structural fingerprints using a literature-based similarity benchmark. J Cheminform. 2016;8:36.

3. Schneider N, Lowe DM, Sayle RA, Landrum GA. Development of a novel fingerprint for chemical reactions and its application to large-scale reaction classification and similarity. J Chem Inf Model. 2015;55:39–53.

4. Todeschini R, Consonni V, Xiang H, Holliday J, Buscema M, P. W. Similarity coefficients for binary chemoinformatics data: overview and extended comparison using simulated and real data sets. J Chem Inf Model. 2012;52:2884–901.

5. Rupp M, Schneider P, Schneider G. Distance phenomena in high-dimensional chemical descriptor spaces: consequences for similarity-based approaches. J Comput Chem. 2009;30:2285–96.

6. Al Khalifa A, Haranczyk M, Holliday J. Comparison of nonbinary similarity coefficients for similarity searching, clustering and compound selection. J Chem Inf Model. 2009;49:1193–201.

7. Willett P. Similarity-based approaches to virtual screening. Biochem Soc Trans. 2003;31:603–6.

8. Riniker S, Landrum G. Open-source platform to benchmark fingerprints for ligand-based virtual screening. J Cheminform. 2013;5:26.

9. Giri V, Sivakumar T, Cho K, Kim T, Bhaduri A. RxnSim: a tool to compare biochemical reactions. Bioinformatics. 2015;31:3712–4.

10. Steinbeck C, Hoppe C, Kuhn S, Floris M, Guha R, Willighagen EL. Recent developments of the chemistry development kit (CDK) - an open-source java library for chemo- and bioinformatics. Curr Pharm Des. 2006;12:2111–20.

11. Rogers D, Hahn M. Extended-connectivity fingerprints. J Chem Inf Model. 2010;50:742–54.

12. Klekota J, Roth FP. Chemical substructures that enrich for biological activity. Bioinformatics [Internet]. 2008;24:2518–25. Available from: http://www.ncbi.nlm.nih.gov/pubmed/18784118

13. Choi S, Cha S, Tappert C. A Survey of Binary Similarity and Distance Measures. J. Syst. Cybern. Informatics. 2010;8:43–8.
